# A novel pathogenic single nucleotide germline deletion in *APC* gene in a four generation Chinese family with familial adenomatous polyposis

**DOI:** 10.1038/s41598-017-10395-x

**Published:** 2017-09-27

**Authors:** Zhao Zhang, Shengyun Liang, Dan Wang, Shengran Liang, Yuwei Li, Bingjie Wang, Tao jiang, Guoru Zhao, Xipeng Zhang, Santasree Banerjee

**Affiliations:** 1 Department of Colorectal Surgery, Tianjin Union Medical Center, Tianjin, 300121 China; 2 Shenzhen Institutes of Advanced Technology, Chinese Academy of Sciences, Shenzhen, 518055 China; 3 Department of Pathology, Tianjin Medical University General Hospital, Tianjin, 300052 China; 4 BGI-Shenzhen, Shenzhen, 518083 China; 5 Department of anorectum, Tianjin people’s hospital, Tianjin, 300121 China; 6 Department of anorectum, People Hospital of Xingtai, Xingtai, 054001 China; 7 Department of General Surgery, Tianjin people’s hospital, Tianjin, 300121 China

## Abstract

Familial adenomatous polyposis (FAP) is an autosomal dominant precancerous condition which is associated with germline mutations of the *APC* gene. Clinically, FAP is characterized by the development of multiple colorectal adenomas or polyps which finally result in colorectal cancer by the 40 years age of the patient, if no surgical interventions have been undertaken. In this study, we present a clinical molecular study of a four generation Chinese family with FAP. Diagnosis of FAP was made on the basis of clinical manifestations, family history and medical (colonoscopy and histopathology) records. Genetic screening of the proband and all affected family members were performed by targeted next-generation sequencing and confirmatory Sanger sequencing. Targeted next generation sequencing identified a germline novel heterozygous single nucleotide deletion [c.3418delC; p.Pro1140Leufs*25] in exon18 of *APC* gene, which segregated with the FAP phenotypes in the proband and in all the affected family members whereas absent in unaffected family members as well as in normal healthy controls of same ethnic origin. Our present study expands the mutational spectrum of APC gene and provides evidence to understand the function of *APC* gene in FAP.

## Introduction

Familial adenomatous polyposis (FAP) [MIM# 175100] is a colorectal cancer predisposition condition manifested with the development of numerous colorectal adenomatous polyps, with an autosomal dominant mode of inheritance. The world-wide incidence of FAP is 3-10/100,000, accounting for approximately 1% of all colorectal cancers (CRC)^1^. However, lack of early detection, proper clinical diagnosis and timely treatment with total proctocolectomy, FAP is inevitably transform to symptomatic colorectal cancer (CRC) with presence of numerous colorectal adenomas or polyps by the fourth decade of life^2,3^. In addition, apart from colorectal adenomas, FAP or CRC patients may also manifested with extracolonic manifestations such as desmoids tumors, congenital hypertrophy of the retinal pigment epithelium (CHRPE), lipomas, osteomas, dental abnormalities, epidermoid cysts and upper gastrointestinal polyps^4^. According to the age of onset of the FAP patient and the number of colorectal adenomatous polyps, FAP is classified into two categories, i.e. the classical FAP (CFAP) with more than 100 colorectal adenomatous polyps and early age of onset and attenuated FAP (AFAP) is manifested with 10–100 colorectal adenomatous polyps and late age of onset^5,6^.

FAP is associated with the germline mutations of the *APC* gene. Moreover, *APC* is a tumor suppressor gene, playing a very significant role in cell adhesion, transcriptional activation, cell migration, and apoptosis^7–10^. APC protein is comprises of eight functional subdomain and regulate cell adhesion, polarization, and migration. The major role of APC protein is to control the level of the β-catenin protein in cytoplasm. Germline mutations of *APC* gene leads to formation of a non-functional or truncated APC protein which results into increase the level of the β-catenin protein in cytoplasm. Elevated level of β-catenin protein in combination with activated transcription factors like Tcf causes aberrant *Wnt* signaling pathway that results into uncontrolled cell proliferation, progression and development of colon cancer^11^. Based on the previous reports, most of the pathogenic *APC* germline mutations are majorly fallen into three types or groups, i.e., nonsense/frameshift mutations, splice sites mutations and deep intronic deletions. Frameshift mutations of the *APC* gene lead to the formation of truncated APC proteins^12^. However, the location of the mutation in the *APC* gene is directly correlated with the phenotypic spectrum of the disease, age of onset and the appearance of extracolonic manifestations in FAP patients.

In present study, in order to understand the genetic cause of FAP in the proband and amongst all the affected members of this four generation Chinese family, we screened a panel of 14 genes (*APC*, *MLH1*, *MSH2*, *MSH6*, *PMS2*, *AXIN2*, *BMPR1A*, *EPCAM*, *MLH3*, *MUTYH*, *PMS1*, *PTEN*, *SMAD4*, *STK11*) associated with colorectal cancer by targeted next-generation sequencing. We identified a novel heterozygous single nucleotide germline deletion [c.3418delC; p.Pro1140Leufs*25] of *APC* gene segregating with FAP phenotype among all the FAP patients in this four generation Chinese family, with autosomal dominant mode of inheritance.

## Results

### Family recruitment and clinical examination

**Figure 1:**
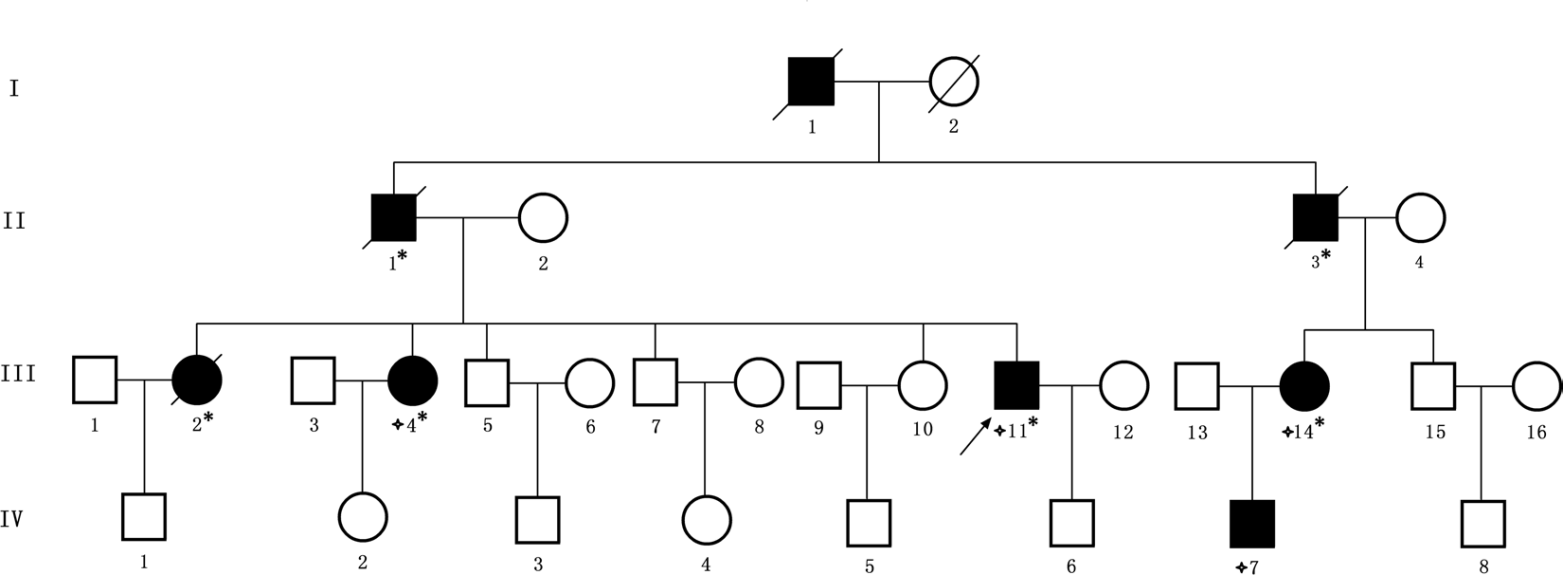
Pedigree structure of the Chinese family with familial adenomatous polyposis. Family members with FAP are indicated with Shading. Squares and circles denoted males and females respectively. Individuals labeled with a solidus were deceased. Family members with CRC are indicated with asterisks. Family members detected with APC gene deletion are indicated by “four angled star”. Roman numerals indicate generations. Arrow indicates the proband (III-11).

We identified a four generation Chinese pedigree with 30 members, among whom four individuals were affected by FAP including three with CRC (Fig. 1). Another 4 affected family members (II-1, II-1, II-3 and III-2) had died from CRC.

### Colonoscopy

**Figure 2:**
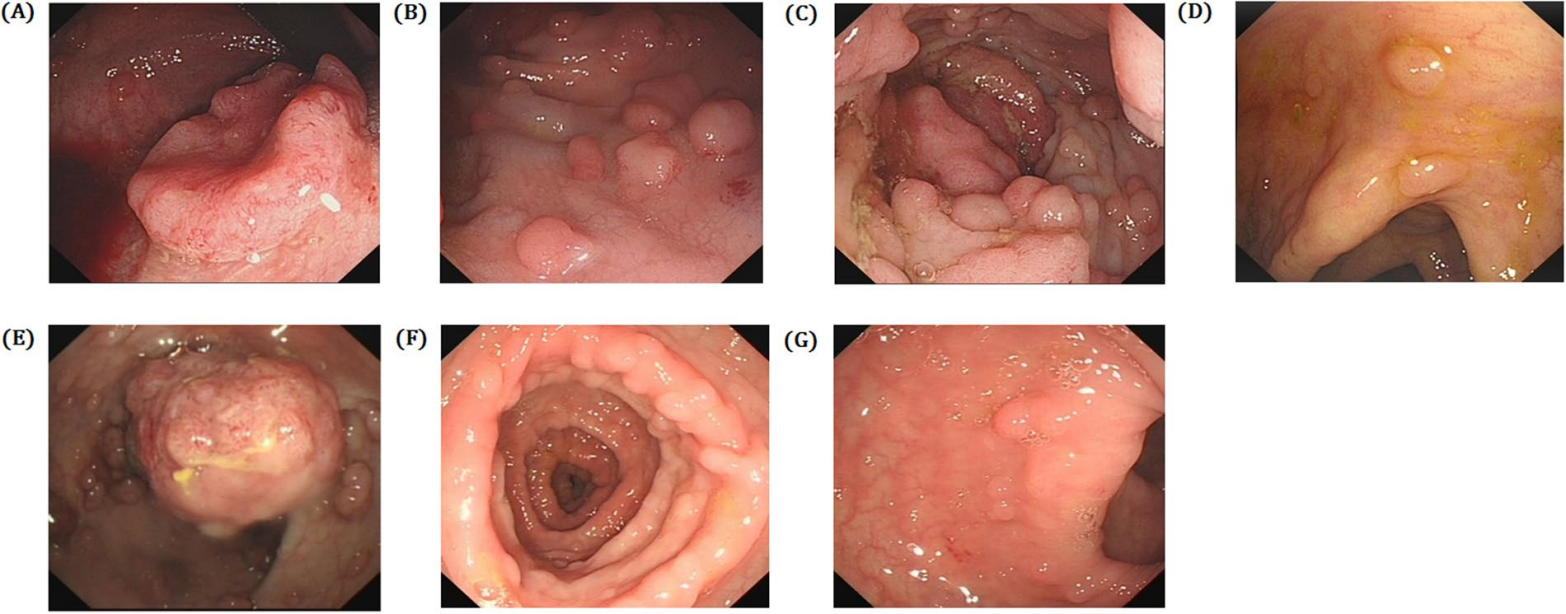
Clinical Description. Colonoscopy of the proband (III-11): (**A**–**C**). There are nearly 100 polyps in sigmoid colon and rectum, the polyps of rectum are malignant transformation, the sigmoid colon is consisting of multiple polyps. (**A**) Lower rectum, (**B**) Sigmoid colon, (**C**) Upper rectum. Colonoscopy of the III-14: (**D**,**E**). There are more than 100 polyps in colon and rectum, the polyps of rectum is transformed into malignant. (**D**) Transverse Colon, (**E**) Rectum. Colonoscopy of IV-7: (**F**,**G**). FAP, there are nearly 100 polps in colorectum, distributed dispersively, maximum diameter is 0.5 cm. (**B**) Transverse Colon, (**G**) Rectum.

Colonoscopy of the proband (III-11) identified nearly 100 polyps in sigmoid colon and rectum the polyps of rectum are transformed into malignant. The sigmoid colon is consisting of multiple polyps (Fig. 2A–C). Colonoscopy of the III-14 showed more than 100 polyps in colon and rectum, the polyps of rectum is transformed into malignant (Fig. 2D,E). Colonoscopy of IV-7 also identified nearly 100 polyps in colon and rectum, distributed dispersive with maximum diameter is 0.5 cm (Fig. 2.F,G).

### Pathology

**Figure 3:**
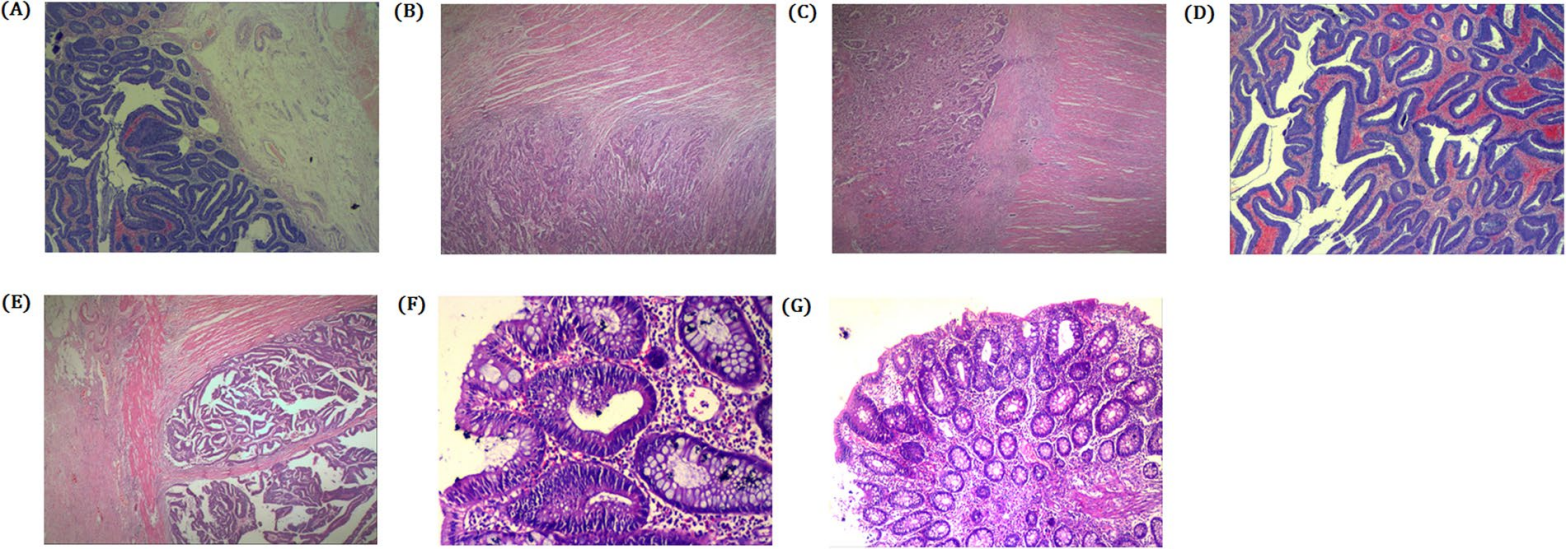
Clinical Description. Pathology of the proband (III-11): (**A**–**C**). (**A**) Transverse colon polyp, low grade intraepithelial neoplasia. (**B**) Lower and moderately differentiated adenocarcinoma of Upper Rectum, Invade the intestinal wall to the perinatal fat, T3N0. (**C**) Moderately differentiated adenocarcinoma of Lower Rectum. Pathology of the III-14: (**D**,**E**). (**D**) Transverse colon polyp, tubular adenoma. (**E**) Polyp of rectum, malignant transformation, moderately differentiated, T3N0. Pathology of the IV-7: (**F**,**G**). (**F**) Rectum, tubular adenoma. (**G**) Sigmoid colon, low grade tubular adenoma.

Pathology of the proband (III-11) identified that low grade intraepithelial neoplasia in the colon. However, lower and moderately differentiated adenocarcinoma of upper rectum. It has been found that moderately differentiated adenocarcinoma of lower rectum (Fig. 3A–C). Pathology of the III-14 identified with polyp in colon with tubular adenoma and also polyp in rectum with malignant transformation (Fig. 3D,E). Pathology of the IV-7 showed that rectum with tubular adenoma and low grade tubular adenoma in the sigmoid colon (Fig. 3F,G).

### Identification and characterization of candidate mutation

A novel heterozygous single nucleotide germline deletion; [c.3418delC; p.Pro1140Leufs*25] in exon18 of APC gene [NCBI Reference sequence NM_000038.3] was identified in proband (III-11) by targeted next generation sequencing. This heterozygous novel deletion co-segregated with the FAP phenotypes in the proband (III-11) and amongst all the affected family (III-4, III-14 and IV-7) members, but absent in the unaffected family members. We did not detect this mutation in the normal control of the same ethnic origin, gender and age range.

**Figure 4:**
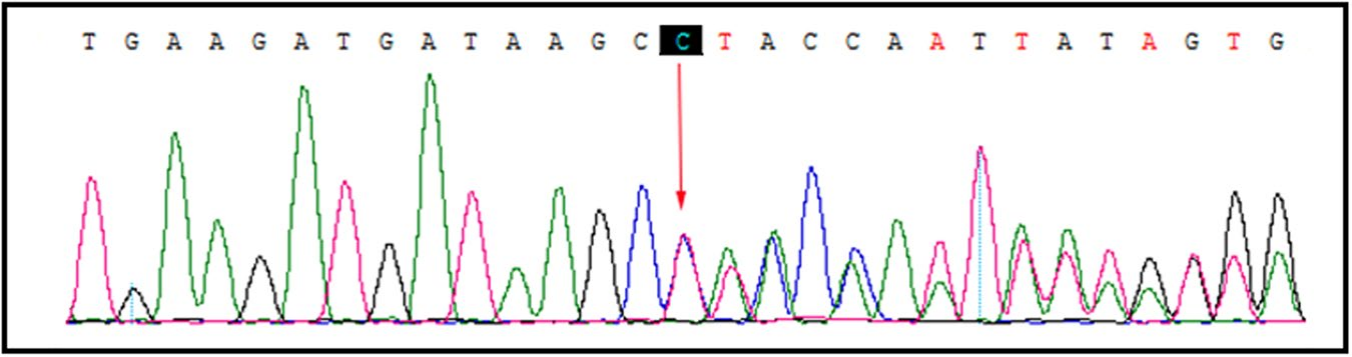
Confirmation of the novel deletion mutation by Sanger sequence.

### Confirmation of the novel large deletion by Sanger Sequencing

All the present (both affected and unaffected) family members are tested for this novel heterozygous deletion mutation [c.3418delC; p.Pro1140Leufs*25] of *APC* gene by Sanger sequencing (Fig. 4).

## Discussion

In our study, we found a heterozygous novel single nucleotide germline deletion [c.3418delC; p.Pro1140Leufs*25] in exon18 of the human *APC* gene in the proband (III-11) and among all the FAP-affected family members (III-4, III-14 and IV-7) in a four generation Chinese family. This heterozygous novel deletion of *APC* gene results in the formation of truncated APC protein. This heterozygous novel large deletion of *APC* gene is not present in the ExAC database.

In this Chinese family, diagnosis of FAP has been done according to the presence of specific clinical symptoms (colorectal adenomas/polyposis) and the autosomal dominant mode of inheritance. In our study, the heterozygous novel large deletion of *APC* gene in this Chinese family manifests with colorectal adenomas (polyps) without any extra-colonic manifestations.

### APC gene mutation

On addition, a huge number of pathogenic *APC* gene mutations have been reported in different population of different countries. According to the HGMD dataset, small deletions account for the majority of *APC* gene mutations, resulting in alteration of the open reading frame followed by the formation of a truncated gene product. Till today, 275 sequence variants of the *APC* gene have been reported in the Chinese population. Among these, 194 are unique variants with identified out of 191 individuals. Among the 275 sequence variants of the APC gene, the majority is substitutions (164), but frameshift (101), nonsense (83), deletion (76), insertion (33) and inversion (2) are also reported (http://www.genomed.org/lovd2/variants_statistics.php).

### Genotype–phenotype correlation

Genotype–phenotype correlation studies are very important and allow us to predict the most likely phenotype to be associated with a given pathogenic mutation. The identification and functional characterization of *APC* mutation carriers with a well diagnosed phenotype will help us to establish specific clinical diagnosis and treatment.

In conclusion, here, we describe a heterozygous novel single nucleotide germline deletion mutation in *APC* gene in a four generation Chinese family with FAP. Our study expands the germline mutational spectrum of the *APC* gene in the Chinese population. Our novel finding contributes to a more comprehensive database of germline mutations of *APC* gene that could be used for the molecular diagnosis, risk assessment, susceptibility of the disease for the FAP patients.

## Materials and Methods

### Ethical statement

Family members of this four generation Chinese family have given written informed consent as they are participating in this study. The Ethical Committee of the Tianjin Union Medical Center, China, reviewed and approved our study protocol in compliance with the Helsinki declaration. Diagnosis of the patients for FAP has done by oncologists, on the basis of clinical test reports and detailed family pedigree.

### Patients and pedigree

A four generation Chinese family with FAP (Fig. 1), diagnosed and treated in the Department of Colorectal Surgery, Tianjin Union Medical Center, 300121, China, were enrolled in our study. Clinical diagnosis of FAP was established in this family by endoscopic screening after the proband (III-11) presented to Tianjin Union Medical Center with CRC. The diagnostic standard or criteria for patients with FAP was as follows: (1) patients having >100 colorectal adenomas or polyps and (2) at least 20 synchronous colorectal adenomas or polyps in patients with a positive family history of FAP.

### Targeted exome-based next-generation sequencing and variant identification

DNA samples obtained from the proband (III-1) were sequenced using target exome-based next-generation sequencing. Roche NimbleGen’s (Madison,USA) custom Sequence Capture Human Array was used to designed to capture 98480 kb of targeted sequence, covering 181 exons and flanking sequence (including the 100 bp of introns) of 14 genes (*APC*, *MLH1*, *MSH2*, *MSH6*, *PMS2*, *AXIN2*, *BMPR1A*, *EPCAM*, *MLH3*, *MUTYH*, *PMS1*, *PTEN*, *SMAD4*, *STK11*) which is associated colorectal cancer (CRC) and yielded an average of 6366534 reads per sample, with approximately 68.78% mapping to the targeted regions. The average sequencing depth of the target area is 464.68 with 99.46% coverage. The procedure for preparation of libraries was consistent with standard operating protocols published previously. In each pooling batch, 10 to 33 samples were sequenced simultaneously on Illumina HiSeq. 2500 Analyzers (Illumina, San Diego, USA) for 90 cycles (specially designed by us for this study). Image analysis, error estimation, and base calling were performed using Illumina Pipeline software (version 1.3.4) to generate raw data. The raw reads were screened to generate–clean reads‖ followed by established filtering criteria. Clean reads with a length of 90 bp were aligned to the reference human genome from the NCBI database (Build 37) using the Burrows Wheeler Aligner (BWA) Multi-Vision software package with output files in–bam‖ format. The bamdata were used for reads coverage in the target region and sequencing depth computation, SNP and INDEL calling, and CNV detection. First, a novel three-step computational frame work for CNV was applied. Then, SNPs and INDELs were called using SOAPsnp software and Sam tools pileup software, respectively. A SNP or INDEL was be filtered if it could not follow the criterion: supported by at least 10 reads and >20% of the total reads. The frequency filter was set at 0.05. If a SNP frequency was more than 0.05 in any of the four databases (dbSNP, Hapmap, 1000 Genomes Project, the 124 healthy reference samples sequenced in this study), it would be regarded as a polymorphism, but not a causative mutation.

Last, SNVs were retrieved in The Human Gene Mutation Database (http://www.hgmd.cf.ac.uk/ac/index.php) and the Leiden Open Variation Database (http://www.lovd.nl/3.0/home), and then labeled as reported or novel.

### Confirmation of the novel deletion mutation by Sanger sequence

To validate true positive of the mutation, Sanger sequencing was performed. Primers flanking the candidate loci were designed based on the reference genomic sequences of Human Genome from GenBank in NCBI and synthesized by Invitrogen, Shanghai, China. PCR amplification was carried out in ABI 9700 Thermal Cycler. PCR products were directly sequenced on ABI PRISM 3730 automated sequencer (Applied Biosystems, Foster City, CA, USA). Sequence data comparisons and analysis were performed by DNASTAR SeqMan (DNASTAR, Madison, Wisconsin, USA).

The heterozygous novel splice-acceptor site mutations identified through targeted next generation sequencing were verified through Sanger sequencing using the primers: F1 5′-TTTACCAGTGAGGGACGGGC-3′, R1 5′-GTTTGTCTGGCTCCGGTAAGTA-3′. The reference sequence NM_000038 of APC was used.
